# Improving coverage measurement for reproductive, maternal, neonatal and child health: gaps and opportunities

**DOI:** 10.7189/jogh.07.010801

**Published:** 2017-06

**Authors:** Melinda K Munos, Cynthia K Stanton, Jennifer Bryce, the Core Group for Improving Coverage Measurement for MNCH

**Affiliations:** 1 Institute for International Programs, Johns Hopkins Bloomberg School of Public Health, Baltimore, MD, USA; 2 Stanton–Hill Research, LLC, Moultonborough, NH, USA; * Individual members are listed in Acknowledgements

## Abstract

**Background:**

Regular monitoring of coverage for reproductive, maternal, neonatal, and child health (RMNCH) is central to assessing progress toward health goals. The objectives of this review were to describe the current state of coverage measurement for RMNCH, assess the extent to which current approaches to coverage measurement cover the spectrum of RMNCH interventions, and prioritize interventions for a novel approach to coverage measurement linking household surveys with provider assessments.

**Methods:**

We included 58 interventions along the RMNCH continuum of care for which there is evidence of effectiveness against cause–specific mortality and stillbirth. We reviewed household surveys and provider assessments used in low– and middle–income countries (LMICs) to determine whether these tools generate measures of intervention coverage, readiness, or quality. For facility–based interventions, we assessed the feasibility of linking provider assessments to household surveys to provide estimates of intervention coverage.

**Results:**

Fewer than half (24 of 58) of included RMNCH interventions are measured in standard household surveys. The periconceptional, antenatal, and intrapartum periods were poorly represented. All but one of the interventions not measured in household surveys are facility–based, and 13 of these would be highly feasible to measure by linking provider assessments to household surveys.

**Conclusions:**

We found important gaps in coverage measurement for proven RMNCH interventions, particularly around the time of birth. Based on our findings, we propose three sets of actions to improve coverage measurement for RMNCH, focused on validation of coverage measures and development of new measurement approaches feasible for use at scale in LMICs.

New calls for investment in reducing mortality among women, newborns and children are welcome [1], especially to the extent that they are tightly focused on delivering interventions of proven effectiveness at high, sustained, and equitable levels of coverage. Also welcome is a new emphasis on accountability in women’s and children’s health [2]. Taken together, the global agendas for reproductive, maternal, newborn, and child health (RMNCH) and for accountability rest on the assumption that country governments and development partners will generate or have access to a minimum set of timely, high–quality, representative data to inform their policy and program decisions.

Regular monitoring of population–based coverage levels for RMNCH is central to assessing progress toward national and international health goals (**Box 1** provides definitions for “intervention coverage” and other terminology used in this paper) [3]. Coverage estimates that guide decisions must provide a valid measure of coverage in a population, be sensitive to changes in program effort, and be reliable across settings and over time. But measuring population–based coverage is not easy (**Box 2**) [5]. A particularly challenging issue is ensuring that the denominator for a coverage indicator is representative of all women or children who need an intervention.

Box 1 **Definition of terms**

**Intervention coverage.** The proportion of a defined population in need of an intervention that actually receive it (usually measured in a probability sample of the population).

**Linking studies.** Studies that link caregivers’ reports of where care was sought with assessments of the interventions delivered by service providers.

**Readiness.** A measure of whether a service provider is prepared to provide an intervention, taking into account the presence of the necessary drugs, commodities, and trained and supervised staff to administer the intervention to individuals in need.

**Quality of care.** A measure of whether an individual in need of an intervention received that intervention from a service provider, including appropriate diagnosis and treatment.

**Reliability.** A measure of whether an indicator provides a consistent measure of population intervention coverage across samples, most typically thought of as the precision of a point estimate.

**Validity.** A measure of whether an indicator provides an unbiased measure of true population intervention coverage.

**Validation study.** An assessment of the extent to which a measure fulfils its intended purpose. This is generally by means of an analytic study which systematically assesses measurement errors and biases and compares data to a “gold standard” or true value, where available.

Box 2 **Key issues in measuring intervention coverage**

These key issues, and other sources of error in survey measurement of intervention coverage, have been extensively discussed by Eisele and colleagues [4].

**Defining the denominator.** The denominator should include only those individuals who are in need of an intervention. These individuals may be identified based on age and/or sex, an event such as pregnancy or childbirth, or a diagnosis of disease. Information error or bias can result in misclassification of individuals as being in (or not in) the denominator.

**Defining the numerator.** The numerator should include individuals who are in need of an intervention and who received that intervention. Information error and bias may affect the identification of individuals in the numerator.

**Information error.** Information error occurs when survey respondents provide a response even when they do not understand the question or do not know the answer, resulting in potential misclassification. Information error is random and increases the variance of a coverage estimate but does not affect the point estimate. The length of the recall period, question wording, and type of information the respondent is asked for can all contribute to information error.

**Information bias.** Information bias occurs when there is systematic error in providing information on the numerator or denominator. It is non–random and can result in under– or over–estimation of the point estimate. Many factors can contribute to information bias, including poor question wording (eg, non–neutral questions), long recall periods leading to recall error or age or date heaping, and the social desirability of one or more of the responses.

There is increasing recognition that caregivers cannot report accurately during a household survey interview about whether they or their child received some interventions, especially when the caregiver does not know the specific clinical details of the intervention (eg, which drug was prescribed). This consideration has led to recommendations that reports of where careseeking occurred, collected through household surveys, be linked to assessments of the interventions provided by service providers in order to support estimates of population coverage (hereafter referred to as a “linking approach” to coverage measurement) [3].

In this article we present an analysis of the current state of coverage measurement for interventions across the RMNCH continuum of care. One objective of this analysis is to identify gaps in coverage measurement and assess the extent to which current approaches to coverage measurement cover the spectrum of RMNCH interventions. A second objective is to determine the RMNCH interventions for which linking approaches are most needed and feasible. We assess both direct measurement of intervention coverage and the measurement of health provider readiness to deliver an intervention and/or the quality of intervention delivery (“quality of care”). We synthesize our findings as a basis for defining gaps and propose action steps to improve the measurement of coverage for MNCH interventions.

## METHODS

### Interventions included in the review

This review focuses on life–saving interventions across the RMNCH continuum of care that are directed against major causes of maternal, newborn, and under–five mortality and stillbirths, and for which there is clear evidence of effectiveness. The list of interventions included in the Global Investment Framework for Women’s and Children’s Health provided a starting point for identifying these interventions [1]. We considered both biomedical interventions and behaviors, such as the practice of exclusive breastfeeding or sleeping under a bednet (often treated as interventions for global monitoring purposes). The “essential newborn care” intervention was broken into its component practices, including thermal care, immediate breastfeeding, and chlorhexidine for umbilical cord cleansing. Water and sanitation interventions were added based on evidence of their effectiveness in reducing under–five morbidity and mortality [6]. The appendix lists the references of published peer–reviewed articles that describe the underlying evidence base. Typically, this evidence is a systematic review of the published literature on effectiveness, but occasionally it is based on consensus among experts, for example where interventions are established in practice and an evaluation of effectiveness has not been conducted, or where the lack of clinical equipoise has led to such evaluations being considered unethical. We consider measurement issues separately for the following groups of interventions: periconceptional (reproductive), antenatal, intrapartum, postnatal, feeding, under–five, and cross–cutting environmental.

### Types of data that are the focus of the review

For each life–saving intervention addressed by this review, we indicate the possible mode(s) of delivery for the intervention (facility–based, community–based, outreach, and/or behavioral), and identify current sources of population–based coverage data and, for facility–based interventions, readiness or quality of care data that could be linked with careseeking data to produce coverage estimates. For coverage measurement, included data sources must provide representative information on both the numerator (individuals in need of an intervention who received it) and denominator (all individuals in need of an intervention).This review only considers population–based data from surveys and other sources that are administered regularly on a large scale (generally at national level) in low– and middle–income countries. More specialized, bespoke surveys (for example, special surveys conducted for effectiveness or efficacy studies) are not included, as these surveys typically provide data for only one country (or more commonly a sub–national area within a country) and are not a useful data source for most countries seeking to track their progress toward RMNCH goals. The review does not address the practical details of survey design such as sampling strategies and detailed sample size issues.

### Population–based coverage data

Household surveys are the major source for population–based intervention coverage data in low– and middle–income countries. These surveys are particularly valuable because they typically seek to interview a representative sample of the population, and thus provide measures of coverage that take into account the entire population and for which uncertainty estimates can be calculated. This review includes only surveys with a representative sampling design that provide data at national scale and at regular intervals, the Demographic and Health Surveys (DHS) and Multiple Indicator Cluster Surveys (MICS) [7,8]. These are the largest international household survey programmes on population and health, and the two main sources for survey–based coverage estimates used in global databases [9]. We consider both survey programmes in this review. DHS has coordinated more than 325 nationally representative surveys in 91 countries since 1985, and MICS has carried out 279 surveys in 109 countries since 1995. Survey questionnaires are defined and revised through consultative processes that include stakeholders at global and country level. Over time, the two survey programmes have included an increasing number of coverage indicators along the continuum of RMNCH, including all the categories addressed by this review. In addition to measuring the coverage of biomedical interventions, the survey programmes measure the prevalence of behaviors such as feeding practices, as well as the coverage of water and sanitation interventions. Both programmes provide estimates for internationally agreed–upon indicators for monitoring progress in RMNCH.

### Assessment of coverage measurement

For each intervention, we first assessed whether it would be theoretically possible for a representative household survey to establish the coverage denominator, ie, the population in need of the intervention. Our assessment was based on the indications for receiving a particular intervention (for example, whether the intervention is to be given to all children within a particular age range or only to children with a particular diagnosis). We considered that for preventive interventions targeted based on age or other conditions (eg, pregnancy), it would generally be possible to establish an appropriate denominator in a household survey, whereas for treatment interventions requiring a diagnosis or recognition of specific symptoms, it would be possible to establish a denominator only for easily recognizable symptoms such as diarrhea.

We reviewed the questionnaires from MICS Round 5 and DHS Phase 6 for each RMNCH intervention to determine whether the surveys provided measures of the numerator and denominator for the coverage indicator. We also noted the reference period for the coverage indicator, that is, the time period over which the indicator is measured and calculated, generally expressed as an interval of time preceding the survey interview.

### Routine health system and program data

Routine data collected via the health system or by implementing programmes may also have some potential for use in estimating RMNCH intervention coverage. Potential advantages of routine data include their availability at a relatively low cost, on a continuous basis, and at facility or district level. In addition, routine data have the potential to provide information on services in greater detail than can be ascertained from respondent recall in household surveys.

However, routine data also have important limitations. Denominators are limited to those who are in contact with the health system, and therefore do not represent the population as a whole. Numerators may be over–counted, especially for services like vitamin A or immunizations that may be delivered both in facilities and through community–based activities or child health days. Many RMNCH indicators of interest are simply not available through routine data, because the numerator, denominator, or both are not collected. Routine health systems in most low– and middle–income countries are also characterized by poor data quality and completeness, and do not include important variables needed to assess equity. Some routine data may be out of date, or may only be updated irregularly. For these reasons, routine data have not been recommended in many settings for tracking key outcome and coverage indicators, and are not considered as a source for intervention coverage data for the purposes of this review.

### Readiness and quality of care data

Data on service provider readiness and quality of care are typically collected through a survey or census of health providers – which may include health centers, referral facilities, and community health workers. We define readiness as the presence of the necessary drugs, commodities, and/or trained and supervised staff to administer the intervention to individuals in need. Measurements of quality require an observation–based assessment of whether an intervention was actually received by individuals in need of the intervention, but readiness variables are often used as proxies for quality. Health provider surveys record information on readiness components, and may also include observations of service provision with or without an independent assessment of the client’s need for the intervention. For this review, we sought to include assessments of the provision of RMNCH interventions that are administered regularly, in multiple countries, and at national scale. We excluded one–time or single–country assessments, as well as special assessments conducted for a specific study. There was substantial variation in the type of data collected by readiness assessments; we included any assessment that collected data on the availability of the necessary drugs and commodities to deliver the interventions in this review.

To identify provider assessments meeting these criteria, we hand–searched a 2009 review of health facility survey methods [10] as well as the presentations from a technical consultation on linking household surveys and provider assessments [11].

We identified five provider assessments that met our inclusion criteria: the World Health Organization (WHO)’s Service Availability and Readiness Assessment (SARA), the DHS Program’s Service Provision Assessment (SPA), MEASURE Evaluation’s Rapid Health Facility Assessments (R–HFA) and Quick Investigation of Quality (QIQ), and WHO’s IMCI quality of care assessments (previously the IMCI–MCE Health Facility Survey) (**Table 1**).

**Table 1:** Data collected through selected provider assessments

	RMNCH, HIV, Tuberculosis, Non–communicable diseases		Child health (curative)		Family planning
**SARA**	**SPA**	**R–HFA**	**IMCI–QoC**	**QIQ**
**Geographic scope**	**Sample or census**	**Sample or census**	**Sample**	**Sample**	**Sample**
**Readiness:**					
Training	*	X	X	*	
Supervision	*	X	X	X	*
Availability of guidelines/tools	†	X		X	X
Availability of drugs/commodities	X	X	X	X	X
**Quality of care:**					
Observation of service provision		X	X	X	X
Re–exam				X	
Exit interview with patient/caregiver		X	X	X	X
**Competency:**					
Case scenarios/vignettes				X	

SARA – Service Availability and Readiness Assessment, SPA – the DHS Program’s Service Provision Assessment (SPA), R–HFA – MEASURE Evaluation’s Rapid Health Facility Assessments, QIQ – Quick Investigation of Quality, IMCI QoC – Integrated Management of Childhood Illness – Quality of Care Assessment

*One health worker in facility is asked to report on training/supervision for all health workers in facility.

†Interviewer asks about availability of guidelines/tools but does not ask to see them.

For each intervention, we reviewed the questionnaires from these provider assessments to determine whether they assessed readiness, observation–based quality of care, or neither. Interventions not able to be measured through provider assessments were excluded. These include non–health sector interventions, such as the availability of improved water sources and improved sanitation, and interventions that are limited to use or ownership of a commodity, such as insecticide–treated bednets (ITNs). In addition, many behaviors do not lend themselves to measurement through provider assessments, although interventions seeking to influence the behavior (eg, counselling on breastfeeding practices) may be amenable to measurement in provider assessments.

### Feasibility for linking study

For each intervention, we assessed the potential to measure population–based intervention coverage through an approach linking household survey data to provider assessment data, at either the individual level or aggregate community level. This approach makes use of population–based data from a household survey to generate a representative estimate of those in need of the intervention (the denominator). The numerator makes use of both population–based data (to estimate the number of individuals who sought care from a particular provider) and data from service provision assessments to determine whether the provider was “ready” to provide the intervention, information that is not available from a household survey. Feasibility of linking was assessed and categorized as highly feasible/potential/infeasible, by considering whether careseeking data for the intervention could be obtained through a household survey, and whether readiness or quality of care for that intervention could be measured through a provider assessment. Interventions for which either careseeking or readiness/quality of care could not be measured were considered infeasible for a linking study. Interventions for which readiness could not be measured but quality of care might be assessed through observation were considered potential candidates for a linking study. Interventions for which both careseeking and readiness could be measured were considered highly feasible candidates for a linking study. For example, magnesium sulfate for treatment of pre–eclampsia/eclampsia was categorized as highly feasible because careseeking (ANC consultations and facility delivery) is measured via household surveys, and readiness to deliver the intervention (availability of magnesium sulfate, dipstick for urine protein/acetic acid and flame for heating, blood pressure apparatus, and trained staff) is currently collected in provider assessments. On the other hand, treatment of neonatal sepsis with antibiotics was categorized as infeasible, because careseeking for neonatal sepsis is not currently measured in household surveys due to the difficulty in establishing a valid denominator (newborns with signs of sepsis) using a survey questionnaire.

## RESULTS

**Table 2** presents the 58 included interventions, organized across the continuum of care, and the current data sources for coverage, readiness, and quality of care for each intervention.

**Table 2:** RMNCH interventions and data sources

Intervention	Mode of delivery	Could household survey establish population in need?	Currently measured in MICS/DHS?	Interventions measured in DHS/MICS	Facility–based interventions		
**Reference period**	**Currently measured in standard provider assessments?, R (readiness), O (observation), N (no)**	**Source of provider data, R (readiness), O (observation)**	**Feasible for linking study**
**Periconceptional:**							
Contraception	Facility, community, outreach	Yes	Yes	3 y/5 y	R, O	SARA (R), SPA (R,O), QIQ (R,O)	Highly feasible
Periconceptional folic acid supplementation	Facility–based	Yes	No		N		Infeasible
Safe abortion services	Facility–based	No	No		N		Infeasible
Post abortion case management	Facility–based	No	No		R		Infeasible
Ectopic pregnancy case management	Facility–based	No	No		N		Infeasible
**Antenatal:**							
Tetanus toxoid vaccine for pregnant women	Facility and outreach	Yes	Yes	2–5 y	R, O	SARA (R), SPA (R,O), R–HFA (R)	Highly feasible
Intermittent preventive treatment of malaria in pregnancy	Facility–based	Yes	Yes	2–5 y	R, O	SARA (R), SPA (R,O), R–HFA (R)	Highly feasible
Syphilis detection and treatment in pregnancy	Facility–based	Yes	No		R, O	SARA (R), SPA (R,O), R–HFA (R)	Highly feasible
Calcium supplementation for prevention and treatment of eclampsia and pre–eclampsia	Facility–based	Yes	No		N		Potential
Multiple micronutrient supplementation	Facility–based	Yes	No		N		Potential
Balanced energy supplementation	Facility and outreach	Yes	No		N		Infeasible
Detection and management of diabetes in pregnancy	Facility–based	No	No		R	SARA (R), SPA (R)	Highly feasible
Pregnant women sleeping under an insecticide–treated bednet	Behavior	Yes	Yes	Last night			
Treatment of malaria in pregnant women	Facility–based	No	No		R	SARA (R), SPA (R)	Infeasible
Management of pre–eclampsia with magnesium sulfate	Facility–based	No	No		R	SARA (R), SPA (R)	Highly feasible
Detection and management of fetal growth restriction	Facility–based	No	No		N		Infeasible
Anti–retroviral therapy for pregnant women	Facility–based	No	No		R	SARA (R), SPA (R)	Highly feasible
Prevention of mother to child transmission of HIV	Facility–based	No	No		R	SARA (R), SPA (R)	Highly feasible
**Intrapartum:**							
Skilled birth attendant	Facility, community (Service contact)	Yes	Yes	2–5 y	R	SARA (R), SPA (R)	Highly feasible
Clean birth practices	Facility, community	Yes	No		R	SARA (R), SPA (R)	Highly feasible
Immediate assessment and stimulation for newborns	Facility, community	Partial	No		R	SARA (R), SPA (R)	Potential
Neonatal resuscitation	Facility–based	No	No		R	SARA (R), SPA (R), R–HFA (R)	Highly feasible
Antibiotics for preterm premature rupture of membranes	Facility–based	No	No		R	SARA (R), SPA (R)	Highly feasible
Antenatal corticosteroids for preterm labor	Facility–based	No	No		R	SARA (R), SPA (R)	Highly feasible*
Magnesium sulfate for eclampsia	Facility–based	No	No		R	SARA (R), SPA (R)	Highly feasible
Active management of the third stage of labor	Facility–based	Yes	No		R	SARA (R), SPA (R), R–HFA (R)	Potential
Induction of labor for 41+ weeks	Facility–based	No	No		N		Potential
**Postnatal:**							
Postnatal visit for moms and for babies	Facility, community (service contact)	Yes	Yes	2–5 y	N		Highly feasible
Immediate initiation of breastfeeding	Behavior occurring in facility or community	Yes	Yes	2–5 y	N		Potential
Thermal care	Facility, community	Yes	Not currently; likely in future		R	SPA (R)	Potential
Clorhexidine for umbilical cord cleansing	Facility, community	Yes	No		R	SARA (R), SPA (R)	Highly feasible
Kangaroo mother care	Facility, community	No	No		R	SPA (R)	Potential
**Feeding:**							
Breastfeeding	Behavior	Yes	Yes	24 h			
Complementary feeding	Behavior	Yes	Yes	24 h			
**Under–five:**							
Vitamin A supplementation	Facility, outreach	Yes	Yes	6 mo	R	SARA (R), SPA (R)	Infeasible
Polio vaccine	Facility, outreach	Yes	Yes	5 y	R	SARA (R), SPA (R)	Infeasible
BCG vaccine	Facility–based	Yes	Yes	5 y	R	SARA (R), SPA (R)	Infeasible
Meningitis vaccine	Facility, outreach	Yes	No		N		Infeasible
Pentavalent3/DPT3 vaccine	Facility–based	Yes	Yes	5 y	R	SARA (R), SPA (R), R–HFA (R)	Infeasible
Pneumococcal vaccine	Facility–based	Yes	Yes	5 y	R	SARA (R)	Infeasible
Rotavirus vaccine	Facility–based	Yes	Yes	5 y	R	SARA (R)	Infeasible
Measles vaccine	Facility, outreach	Yes	Yes	5 y	R	SARA (R), SPA (R), R–HFA (R)	Infeasible
Antibiotics for neonatal sepsis	Facility, community	No	No		R	SARA (R), SPA (R), R–HFA (R)	Infeasible
Oral rehydration solution for diarrhea	Facility, community	Yes	Yes	2 weeks	R, O	SARA (R), SPA (R,O), IMCI (R,O), R–HFA (R,O)	Highly feasible†
Zinc for diarrhea	Facility, community	Yes	Yes	2 weeks	R, O	SARA (R), SPA (R,O), IMCI (R,O)	Highly feasible
Antibiotics for dysentery	Facility–based	No	No		R, O	SARA (R), SPA (R,O), IMCI (R,O), R–HFA (R,O)	Highly feasible
Antibiotics for suspected pneumonia	Facility, community	No	Yes	2 weeks	R, O	SARA (R), SPA (R,O), IMCI (R,O), R–HFA (R,O)	Highly feasible
Artemisinin combination therapies for malaria	Facility, community	No	Yes	2 weeks	R, O	SARA (R), SPA (R,O), IMCI (R,O), R–HFA (R,O)	Highly feasible
Vitamin A treatment for measles	Facility–based	No	No		R, O	SARA (R), SPA (R,O), IMCI (R,O), R–HFA (R)	Infeasible
Management of severe malnutrition	Facility–based	No	No		N		Infeasible
Cotrimoxazole for HIV	Facility–based	No	No		R	SARA (R), SPA (R)	Infeasible
Paediatric anti–retroviral therapy for HIV	Facility–based	No	No		R	SARA (R), SPA (R)	Infeasible
**Environmental:**							
Use of improved water source	Behavior	Yes	Yes	NA			
Use of improved sanitation	Behavior	Yes	Yes	NA			
Hygienic disposal of children's stool	Behavior	Yes	Yes	Last stool			
Handwashing	Behavior	Yes	No				
Insecticide–treated bednet ownership	Outreach	Yes	Yes	NA			
Insecticide–treated bednet use	Behavior	Yes	Yes	Last night			

SARA – Service Availability and Readiness Assessment, SPA – the DHS Program’s Service Provision Assessment (SPA), R–HFA – MEASURE Evaluation’s Rapid Health Facility Assessments, QIQ – Quick Investigation of Quality, IMCI – WHO integrated management of childhood illness (previously the IMCI–MCE Health Facility Survey)

*A recent study has called into question the benefits of antenatal corticosteroids in low– and middle–income countries [12].

†In settings where ORS is primarily distributed through health facilities and community health workers.

### Measurement of intervention coverage through household surveys

Twenty–four, or fewer than half, of the included interventions are currently measured through regular household surveys (DHS or MICS). Of those interventions not measured through household surveys, all but one (handwashing) are delivered at health facilities; five can also be delivered at community level and two via outreach. Two of the measured interventions are proxies for intervention coverage and actually measure careseeking, ie, skilled birth attendance and postnatal visits, rather than interventions. Many of the measured interventions fall in the under–five and environmental categories, with 11 of 18 under–five interventions and five of six environmental interventions measured in MICS and DHS. Within the under–five category, however, there are gaps with respect to measuring treatment of malnutrition and neonatal infections. Along the continuum of care, the intrapartum period stands out as the highest risk period for women and babies, and yet none of the included interventions for this period is measured in surveys beyond service contacts. Similarly, a relatively low proportion of antenatal (two of 13) and periconceptional (one of five) interventions are measured through MICS and DHS.

### Measurement of readiness and quality of care

Of the 49 interventions that can be delivered at a health facility, provider assessments currently measure readiness for 27 interventions, and readiness and observation–based quality of care for 10 interventions. Those interventions not currently addressed by provider assessments are primarily periconceptional and antenatal in nature (for example, safe abortion services, calcium supplementation, and detection and management of fetal growth restriction). The WHO’s SARA and the DHS Program’s SPA are the main sources of these data. These two assessments provide data for most of the same interventions. SPAs provide a more complete assessment of health worker training and supervision, as well as the quality of services.

### Feasibility of measuring coverage through linked provider assessments and household surveys

Estimating intervention coverage using a linked approach requires the ability to measure careseeking through a population–based household survey and provider readiness to deliver the intervention (or quality of delivery of the intervention) through a health provider assessment. These two sources of information then must be linked, either by matching each individual in the household survey to a particular facility, or by associating everyone in the household survey within a catchment area to a particular facility. We estimate that a linking approach would be highly feasible for 22 interventions, 13 of which are not currently measured in household surveys – five antenatal, six intrapartum, one postnatal, and one under–five intervention. For another five intrapartum and postnatal indicators, a linking approach might be feasible if observation–based provider assessments were used.

## DISCUSSION

Given the increasing global attention to accountability for RMNCH and awareness of the importance of intervention coverage to achieve mortality reductions, there is a critical need to measure population coverage of life–saving RMNCH interventions at national scale and on a regular basis. This review sought to map out which interventions are currently measured, and by what means, in order to identify gaps in current approaches to coverage measurement, and to assess the potential for using a new approach linking household surveys and provider assessments to provide estimates of intervention coverage.

A positive finding of this review is that many interventions targeted to children aged 1–59 months are currently measured through large, nationally representative household surveys, as are many environmental interventions. Beyond child health and environmental interventions, however, we found that many lifesaving interventions in the periconceptional, antenatal, intrapartum, and postnatal periods are not currently measured through population–based household surveys. Although some of these interventions may be measured through routine or program data, such data often lack an appropriate denominator and have issues of data quality and completeness. However, we also found that many antenatal and intrapartum interventions are currently measured through provider assessments and would be good candidates for measurement through an approach linking household surveys to provider assessments.

### Gaps in coverage measurement

In general, we found that household surveys are not good sources of coverage data for interventions that require caregiver or respondent knowledge of specific clinical details such as a diagnosis. The exception is conditions for which biomarkers are available. Although new biomarker tests are increasingly available, their use in large–scale surveys is restricted to a few indicators and, where they are available, their use can be complicated and expensive. Household surveys are generally well–suited for measuring preventive interventions and assessing careseeking based on symptoms that can easily be recognized and recalled by mothers. There is a clear measurement gap for interventions delivered during pregnancy and around the time of birth. Household surveys primarily measure careseeking for these periods, and therefore cannot currently be used to track progress in the coverage of most reproductive, maternal, and neonatal interventions. Moreover, many of these interventions are not appropriate for measurement through household surveys, because they require a diagnosis, such as pre–eclampsia or preterm premature rupture of membranes, which cannot be readily established through a survey questionnaire. Household surveys are also not suited to measuring coverage of interventions needed by very small numbers of individuals (such as antibiotics for preterm premature rupture of membranes), as household surveys typically cannot achieve adequate sample sizes to provide precise coverage estimates, for these intervnetions. This gap is of particular concern given the importance of the period around and immediately after birth for the health of mothers and babies: most maternal and newborn deaths occur during childbirth and in the day following birth [13], and neonatal deaths represent a growing proportion of under–five deaths [14]. Tracking the coverage of interventions that protect against common causes of maternal and neonatal deaths is thus critical to ensuring progress in RMNCH, and is not possible at present.

Another important gap is the lack of data on the accuracy [3], precision, and reliability of the coverage data collected through household surveys. Where data on indicator validity exist, they suggest that although household surveys can provide accurate coverage measures for some interventions, such as treatment of fever with an ACT [15], other interventions such as antibiotics for pneumonia are not well measured through such surveys [16]. The question of whether coverage measurements are reliable over time and across countries is of central importance if survey data are to be used to track progress in coverage of RMNCH interventions. There is an urgent need for research to better understand which health interventions household surveys can provide accurate, precise, and reliable population–based coverage measures, and for which interventions alternative measurement approaches should be explored. A few recent studies have explored the validity of a range of coverage measures for the intrapartum and immediate postnatal period with mixed results [17]. A clear alternative to measuring careseeking (ie, skilled birth attendance) has not yet emerged for the intrapartum period.

### Limitations

This review has a number of limitations. Our list of interventions was based on those in the Global Investment Framework for Women’s and Children’s Health, and included only interventions with published effectiveness estimates (see **Appendix S1** in **Online Supplementary Document(Online Supplementary Document)**). However, there may be interventions, particularly emerging interventions for which the body of evidence is still developing, that have been omitted. As new interventions emerge over time, there will be an ongoing need to consider whether and how to measure their coverage.

Our process for assessing the feasibility of using a linking approach to estimate the coverage of each intervention was somewhat subjective. Although we attempted to establish clear criteria for each level of feasibility, it is possible that another group might come to somewhat different conclusions. There are ongoing efforts to implement the linking approach using existing and new data. When complete, these studies will provide additional information about the feasibility of linking for various interventions.

Finally, we note that household and provider surveys and routine data continue to evolve. This review provides a snapshot of the gaps and opportunities at a particular point in time. We expect that some of the gaps identified here will be filled over time as data collection instruments are revised and routine health information systems improve.

### Research and practice agendas

Providing valid, population–based estimates of coverage for RMNCH interventions at national and sub–national levels is essential to achieving reductions in maternal, newborn, and child deaths and stillbirths, and must be a priority for the RMNCH research and practice community. We recommend three parallel streams of action to improve the availability and quality of data on intervention coverage for RMNCH.

#### Action stream 1: household surveys

Household surveys should continue to be used as a source of coverage data for those indicators that can be measured through a survey questionnaire. Efforts to validate survey–based measures of RMNCH intervention coverage must continue and must include assessments of the reliability of coverage measurements over time. The results of these efforts should inform future revisions of MICS and DHS survey questionnaires. Where the evidence indicates that surveys do not provide accurate, precise, or reliable measures of intervention coverage, alternative measurement approaches should be explored.

#### Action stream 2: alternative measurement approaches for facility–based interventions

Many of the RMNCH interventions not measured in household surveys, including those that cannot be measured in a household survey because they require a diagnosis, are delivered by a health service provider, and are currently measured in provider assessments. Measurement approaches that link these service provider assessments to data on careseeking collected through household surveys must be pursued urgently. Linking approaches could also be valuable for indicators currently measured in surveys, but for which the validity of the survey–based indicator is questionable, including treatment of childhood illness. Assessments of linking approaches should address the following factors: feasibility and cost at national scale in low– and middle–income countries, as well as the accuracy and reliability of coverage measures produced through this approach. In addition, different approaches to linking household surveys and provider assessments should be tested and compared.

Other approaches to measuring coverage for facility–based interventions, including the use of routine data, may also hold promise and should be assessed using the same considerations as linking approaches (feasibility, cost, accuracy, and reliability).

For approaches that are found to be feasible to implement at reasonable cost and to provide both accurate and reliable measures of intervention coverage, the RMNCH research and practice community should develop guidelines for their implementation and a program to ensure the regular production of coverage measures for these interventions.

#### Action stream 3: alternative measurement approaches for non–facility–based interventions

For those interventions for which household surveys do not provide accurate or reliable measurements, and which are delivered primarily or entirely outside a facility, a linking a pproach is not feasible and alternative measurement approaches, such as the use of specialized surveys, biomarkers or proxies for the intervention, or modeling, should be explored. This is true for behaviors as well, although a linking approach should be explored for interventions promoting the behavior, such as counselling on breastfeeding practices.

## References

[1] Stenberg K, Axelson H, Sheehan P, Anderson I, Gülmezoglu AM, Temmerman M (2014). Advancing social and economic development by investing in women’s and children’s health: a new Global Investment Framework.. Lancet.

[2] World Health Organization. Commission on Information and Accountability for Women's and Children's Health. Available: http://www.who.int/woman_child_accountability/about/coia/en/ Accessed: 4 Apr 2017.

[3] Bryce J, Arnold F, Blanc A, Hancioglu A, Newby H, Requejo J (2013). Measuring coverage in MNCH: new findings, new strategies, and recommendations for action.. PLoS Med.

[4] Eisele TP, Rhoda DA, Cutts FT, Keating J, Ren R, Barros AJ (2013). Measuring coverage in MNCH: total survey error and the interpretation of intervention coverage estimates from household surveys.. PLoS Med.

[5] PLoS Collections. Measuring Coverage in Maternal, Newborn, and Child Health. 2013. Available: http://www.ploscollections.org/measuringcoverageinmnch. Accessed 13 January 2015.

[6] Cairncross S, Hunt C, Boisson S, Bostoen K, Curtis V, Fung IC (2010). Water, sanitation and hygiene for the prevention of diarrhoea.. Int J Epidemiol.

[7] International ICF. Demographic and Health Surveys. Available: http://www.dhsprogram.com Accessed: 3 May 2014.

[8] UNICEF. Statistics and Monitoring: Multiple Indicator Cluster Survey. Available: http://www.unicef.org/statistics/index_24302.html. Accessed: 3 May 2014.

[9] Hancioglu A, Arnold F (2013). Measuring coverage in MNCH: tracking progress in health for women and children using DHS and MICS household surveys.. PLoS Med.

[10] Edward A, Matsubiyashi T, Fapohunda B, Becker S. A Comparative Analysis of Select Health Facility Survey Methods Applied in Low and Middle Income Countries [working paper WP–09–11]. Chapel Hill, NC: 2009.

[11] Institute for International Programs. Linking Household Surveys and Health Service Assessments: Technical Consultation on Study Design. Baltimore, MD: 2014.

[12] Althabe F, Belizán JM, McClure EM, Hemingway–Foday J, Berrueta M, Mazzoni A (2015). A population–based, multifaceted strategy to implement antenatal corticosteroid treatment versus standard care for the reduction of neonatal mortality due to preterm birth in low–income and middle–income countries: the ACT cluster–randomised trial.. Lancet.

[13] Lawn JE, Blencowe H, Oza S, You D, Lee AC, Waiswa P (2014). Every Newborn: progress, priorities, and potential beyond survival.. Lancet.

[14] Liu L, Oza S, Hogan D, Perin J, Rudan I, Lawn JE (2015). Global, regional, and national causes of child mortality in 2000–13, with projections to inform post–2015 priorities: an updated systematic analysis.. Lancet.

[15] Eisele TP, Silumbe K, Yukich J, Hamainza B, Keating J, Bennett A (2013). Measuring coverage in MNCH: accuracy of measuring diagnosis and treatment of childhood malaria from household surveys in Zambia.. PLoS Med.

[16] Hazir T, Begum K, El Arifeen S, Khan AM, Huque MH, Kazmi N (2013). Measuring coverage in MNCH: a prospective validation study in Pakistan and Bangladesh on measuring correct treatment of childhood pneumonia.. PLoS Med.

[17] Stanton CK, Rawlins B, Drake M, Dos Anjos M, Cantor D, Chongo L (2013). Measuring coverage in MNCH: testing the validity of women’s self–report of key maternal and newborn health interventions during the peripartum period in Mozambique.. PLoS One.

