# Supplementary material for: Improving coverage measurement for reproductive, maternal, neonatal and child health: gaps and opportunities
Source: J Glob Health. 2017 Jun 4;7(1):010801. doi: 10.7189/jogh.07.010801 (PMC5460400; doi:10.7189/jogh.07.010801)
Supplement: Online Supplementary Document [file jogh-07-010801-s001.pdf]

Online Supplementary Document

Munos et al. Improving coverage measurement for reproductive, maternal, neonatal and child health: gaps and opportunities

J Glob Health 2017;7:010801

Appendix 1

Periconceptual interventions

Adler AJ, Filippi V, Thomas SL, Ronsmans C. Quantifying the global burden of morbidity due to unsafe abortion: magnitude in hospital-based studies and methodological issues. Int J Gynaecol Obstet 2012; 118 Suppl 2: S65-77.

Ahmed S, Li Q, Liu L, Tsui AO. Maternal deaths averted by contraceptive use: an analysis of 172 countries. Lancet 2012; 380(9837): 111-25.

Blencowe H, Cousens S, Modell B, Lawn J. Folic acid to reduce neonatal mortality from neural tube disorders. Int J Epidemiol 2010; 39 Suppl 1: i110-21.

Pollard SL, Mathai M, Walker N. Estimating the impact of interventions on cause-specific maternal mortality: a Delphi approach. BMC Public Health 2013; 13(3): 1.

Antenatal interventions

Blencowe H, Lawn J, Vandelaer J, Roper M, Cousens S. Tetanus toxoid immunization to reduce mortality from neonatal tetanus. Int J Epidemiol 2010; 39 Suppl 1: i102-9.

Blencowe H, Cousens S, Kamb M, Berman S, Lawn JE. Lives Saved Tool supplement detection and treatment of syphilis in pregnancy to reduce syphilis related stillbirths and neonatal mortality. BMC Public Health 2011; 11 Suppl 3: S9.

Eisele TP, Larsen DA, Anglewicz PA, et al. Malaria prevention in pregnancy, birthweight, and neonatal mortality: a meta-analysis of 32 national cross-sectional datasets in Africa. Lancet Infect Dis 2012; 12(12): 942-9.

Haider BA, Yakoob MY, Bhutta ZA. Effect of multiple micronutrient supplementation during pregnancy on maternal and birth outcomes. BMC Public Health 2011; 11 Suppl 3: S19.

Imdad A, Bhutta ZA. Effect of balanced protein energy supplementation during pregnancy on birth outcomes. BMC Public Health 2011; 11 Suppl 3: S17.

Imdad A, Jabeen A, Bhutta ZA. Role of calcium supplementation during pregnancy in reducing risk of developing gestational hypertensive disorders: a meta-analysis of studies from developing countries. BMC Public Health 2011; 11 Suppl 3: S18.

Imdad A, Yakoob MY, Bhutta ZA. The effect of folic acid, protein energy and multiple micronutrient supplements in pregnancy on stillbirths. BMC Public Health 2011; 11 Suppl 3: S4.

Imdad A, Yakoob MY, Siddiqui S, Bhutta ZA. Screening and triage of intrauterine growth restriction (IUGR) in general population and high risk pregnancies: a systematic review with a focus on reduction of IUGR related stillbirths. BMC Public Health 2011; 11 Suppl 3: S1

Ishaque S, Yakoob MY, Imdad A, Goldenberg RL, Eisele TP, Bhutta ZA. Effectiveness of interventions to screen and manage infections during pregnancy on reducing stillbirths: a review. BMC Public Health 2011; 11 Suppl 3: S3.

Jabeen M, Yakoob MY, Imdad A, Bhutta ZA. Impact of interventions to prevent and manage preeclampsia and eclampsia on stillbirths. BMC Public Health 2011; 11 Suppl 3: S6.

Pollard SL, Mathai M, Walker N. Estimating the impact of interventions on cause-specific maternal mortality: a Delphi approach. BMC Public Health 2013; 13(3): 1.

Ronsmans C, Campbell O. Quantifying the fall in mortality associated with interventions related to hypertensive diseases of pregnancy. BMC Public Health 2011; 11 Suppl 3: S8.

Syed M, Javed H, Yakoob MY, Bhutta ZA. Effect of screening and management of diabetes during pregnancy on stillbirths. BMC Public Health 2011; 11 Suppl 3: S2.

Intrapartum interventions

Blencowe H, Cousens S, Mullany LC, et al. Clean birth and postnatal care practices to reduce neonatal deaths from sepsis and tetanus: a systematic review and Delphi estimation of mortality effect. BMC Public Health 2011; 11 Suppl 3: S11.

Cousens S, Blencowe H, Gravett M, Lawn JE. Antibiotics for pre-term pre-labour rupture of membranes: prevention of neonatal deaths due to complications of pre-term birth and infection. Int J Epidemiol 2010; 39 Suppl 1: i134-43.

Hussain AA, Yakoob MY, Imdad A, Bhutta ZA. Elective induction for pregnancies at or beyond 41 weeks of gestation and its impact on stillbirths: a systematic review with meta-analysis. BMC Public Health 2011; 11(3): 1.

Lee AC, Cousens S, Darmstadt GL, et al. Care during labor and birth for the prevention of intrapartum-related neonatal deaths: a systematic review and Delphi estimation of mortality effect. BMC Public Health 2011; 11 Suppl 3: S10.

Lee AC, Cousens S, Wall SN, et al. Neonatal resuscitation and immediate newborn assessment and stimulation for the prevention of neonatal deaths: a systematic review, meta-analysis and Delphi estimation of mortality effect. BMC Public Health 2011; 11 Suppl 3: S12.

Mwansa-Kambafwile J, Cousens S, Hansen T, Lawn JE. Antenatal steroids in preterm labour for the prevention of neonatal deaths due to complications of preterm birth. Int J Epidemiol 2010; 39 Suppl 1: i122-33.

Pollard SL, Mathai M, Walker N. Estimating the impact of interventions on cause-specific maternal mortality: a Delphi approach. BMC Public Health 2013; 13(3): 1.

Yakoob MY, Ali MA, Ali MU, et al. The effect of providing skilled birth attendance and emergency obstetric care in preventing stillbirths. BMC Public Health 2011; 11 Suppl 3: S7.

Postnatal interventions

Debes AK, Kohli A, Walker N, Edmond K, Mullany LC. Time to initiation of breastfeeding and neonatal mortality and morbidity: a systematic review. BMC Public Health 2013; 13 Suppl 3: S19.

Imdad A, Mullany LC, Baqui AH, et al. The effect of umbilical cord cleansing with chlorhexidine on omphalitis and neonatal mortality in community settings in developing countries: a meta-analysis. BMC Public Health 2013; 13 Suppl 3: S15.

Lawn JE, Mwansa-Kambafwile J, Horta BL, Barros FC, Cousens S. 'Kangaroo mother care'to prevent neonatal deaths due to preterm birth complications. International journal of epidemiology 2010; 39(suppl 1): i144-i54.

Feeding practices and interventions

Haroon S, Das JK, Salam RA, Imdad A, Bhutta ZA. Breastfeeding promotion interventions and breastfeeding practices: a systematic review. BMC Public Health 2013; 13 Suppl 3: S20.

Imdad A, Yakoob MY, Bhutta ZA. Impact of maternal education about complementary feeding and provision of complementary foods on child growth in developing countries. BMC Public Health 2011; 11(3): 1.

Imdad A, Yakoob MY, Bhutta ZA. Effect of breastfeeding promotion interventions on breastfeeding rates, with special focus on developing countries. BMC Public Health 2011; 11 Suppl 3: S24.

Lamberti LM, Zakarija-Grkovic I, Fischer Walker CL, et al. Breastfeeding for reducing the risk of pneumonia morbidity and mortality in children under two: a systematic literature review and meta-analysis. BMC Public Health 2013; 13 Suppl 3: S18.

Lamberti LM, Fischer Walker CL, Noiman A, Victora C, Black RE. Breastfeeding and the risk for diarrhea morbidity and mortality. BMC Public Health 2011; 11 Suppl 3: S15.

Lassi ZS, Das JK, Zahid G, Imdad A, Bhutta ZA. Impact of education and provision of complementary feeding on growth and morbidity in children less than 2 years of age in developing countries: a systematic review. BMC Public Health 2013; 13 Suppl 3: S13.

Under-five interventions

Das JK, Ali A, Salam RA, Bhutta ZA. Antibiotics for the treatment of Cholera, Shigella and Cryptosporidium in children. BMC Public Health 2013; 13 Suppl 3: S10.

Walker CLF, Black RE. Zinc for the treatment of diarrhoea: effect on diarrhoea morbidity, mortality and incidence of future episodes. International journal of epidemiology 2010; 39(suppl 1): i63-i9.

Imdad A, Yakoob MY, Sudfeld C, Haider BA, Black RE, Bhutta ZA. Impact of vitamin A supplementation on infant and childhood mortality. BMC Public Health 2011; 11 Suppl 3: S20.

Munos MK, Walker CL, Black RE. The effect of oral rehydration solution and recommended home fluids on diarrhoea mortality. Int J Epidemiol 2010; 39 Suppl 1: i75-87.

Munos MK, Walker CL, Black RE. The effect of rotavirus vaccine on diarrhoea mortality. Int J Epidemiol 2010; 39 Suppl 1: i56-62.

Sudfeld CR, Navar AM, Halsey NA. Effectiveness of measles vaccination and vitamin A treatment. Int J Epidemiol 2010; 39 Suppl 1: i48-55.

Theodoratou E, Al-Jilaihawi S, Woodward F, et al. The effect of case management on childhood pneumonia mortality in developing countries. Int J Epidemiol 2010; 39 Suppl 1: i155-71.

Theodoratou E, Johnson S, Jhass A, et al. The effect of Haemophilus influenzae type b and pneumococcal conjugate vaccines on childhood pneumonia incidence, severe morbidity and mortality. Int J Epidemiol 2010; 39 Suppl 1: i172-85.

Thwing J, Eisele TP, Steketee RW. Protective efficacy of malaria case management and intermittent preventive treatment for preventing malaria mortality in children: a systematic review for the Lives Saved Tool. BMC Public Health 2011; 11 Suppl 3: S14.

Traa BS, Walker CL, Munos M, Black RE. Antibiotics for the treatment of dysentery in children. Int J Epidemiol 2010; 39 Suppl 1: i70-4.

Zaidi AK, Ganatra HA, Syed S, et al. Effect of case management on neonatal mortality due to sepsis and pneumonia. BMC Public Health 2011; 11(3): 1.

Environmental interventions

Cairncross S, Hunt C, Boisson S, et al. Water, sanitation and hygiene for the prevention of diarrhoea. Int J Epidemiol 2010; 39 Suppl 1: i193-205.

Eisele TP, Larsen D, Steketee RW. Protective efficacy of interventions for preventing malaria mortality in children in Plasmodium falciparum endemic areas. Int J Epidemiol 2010; 39 Suppl 1: i88-101.
